# The Network of Antigen-Antibody Reactions in Adult Women with Breast Cancer or Benign Breast Pathology or without Breast Pathology

**DOI:** 10.1371/journal.pone.0119014

**Published:** 2015-03-17

**Authors:** Tania Romo-González, Marcela Esquivel-Velázquez, Pedro Ostoa-Saloma, Carlos Lara, Alejandro Zentella, Rosalba León-Díaz, Edmundo Lamoyi, Carlos Larralde

**Affiliations:** 1 Instituto de Investigaciones Biológicas, Universidad Veracruzana, Xalapa, Veracruz, México; 2 Programa de Investigación de Cáncer de Mama, Instituto de Investigaciones Biomédicas, Universidad Nacional Autónoma de México, Distrito Federal, México; 3 Hospital General de México, Secretaría de Salubridad y Asistencia, Distrito Federal, México; Weizmann Institute of Science, ISRAEL

## Abstract

The Immunoglobulin G (IgG) antibody response to different protein antigens of the mammary ductal carcinoma by adult women affected by Breast Cancer (BC) distinguishes at least 103 proteins that differ in their molecular weights (MW). The IgG producing cell clones (nodes) coexist with each other in each individual organism and share energy resources among themselves, as well as factors that control the level of expression and Specificity of their IgG antibodies. So, it can be proposed that among them there is a Network of interconnections (links) unveiled by the antigens, which specifically react with the IgG antibodies produced by the clones. This Network possibly regulates IgG antibodies' activity and effectiveness. We describe the Network of nodes and links that exists between the different antigens and their respective IgG producing cell clones against the extracted protein antigens from the cells of the T47D Cell-Line, in 50 women with BC, 50 women with Benign Breast Pathology (BBP) and 50 women without breast pathology (H). We have found that women with BBP have the highest number of Links, followed by the H group and, lastly, the women with BC, a finding which suggests that cancer interferes with the Connectivity between the IgG producing cell clones and blocks the expression of 322 links in women with BBP and 32 links in women with H. It is also plausible that the largest number of links in the women with BBP indicates the Network’s state of arousal that provides protection against BC. On the other hand, there were many missing links in the BC group of women; the clone which lost more links in the BC group was the hub 24, which point to some of the antigens of T47D as potentially useful as vaccines, as the immune system of women with BBP is well aware of them.

## Introduction

Breast Cancer (BC) is the most common cancer in women worldwide, with nearly 1,380,000 diagnostics per year [1]. In 2008, 460,000 deaths were registered, of which 269,000 (58%) were in low and middle-income countries, and 68,000 (15%) occurred in women between 15–49 years of age in low-income countries [2,3]. The highest incidence and mortality of BC in these countries, as well as its appearance at an earlier age, occurs primarily because women of low and middle income countries are not fully incorporated by the prevention programs of BC, nor go to health service centers to perform diagnostic studies (physical examination, ultrasound, mammography) with the recommended frequency, either by fear, indolence, poverty or modesty (on the part of themselves or of their husbands) [4]. That is why, in these same countries, BC is rarely detected in its early stages and the life expectancy of women with BC is, consequently, much lower than that of women with BC in high income countries [3].

As in other middle-income countries, in Mexico cancer mortality has been on the increase: 58 per 100,000 inhabitants in 1998 to 67 per 100,000 in 2008. In addition, since 2006 BC has been the main cause of cancer death among Mexican women, representing 14% of those deaths [5,6]. Globocan predicts that in 2030, 24,386 women will be diagnosed with BC, and 9,778 (40%) will die from BC in Mexico, which makes BC a major challenge for Mexico’s Health System [2].

The detection of BC is usually performed by mammography. However, the number of Mammographers is scarce and there is not a sufficient number of specialized radiologists to interpret the Mammographies [7–9]. In addition, hopes that Mammographies can detect stages of early BC are low, since it has been reported that a breast cancer cell generally duplicates every 100–300 days. As such, a breast tumor of 7 mm in diameter has had to perform about 21 duplications before reaching this size, meaning that a breast tumor visible in a mammography has been evolving for at least 7 years [8,10].

Hence, there is a need to find new and more effective ways to diagnose BC, which are economically affordable, safe, sensitive and easily performed in most clinical laboratories. The more so since there are reports that BC can be detected by the immune system seven years before a positive Mammography [11–13], and several research groups have made efforts to find antibodies and antigens useful for this purpose but, until now there is no serological test accessible to standard clinical laboratories or specific to the Mexican population. In addition, although some of the antigens very specific to BC have been purified up until to now, they are not detectable in most of the sera from women in early stages of BC (*i*.*e*., AutoAntibodies (AAb) to NYESO1 are found in 4%, SCP1in 6%, and SSX2 in only 1% of sera from breast cancer patients) [14].

Our research group has corroborated by Uni-dimensional Immunoblot Images (1D-II) that, indeed, women’s immune systems (IgGs) perceive the protein antigens of the T47D Cell-Line. Using sera from 50 women with ductal breast cancer (BC), 50 women with benign breast pathology (BBP) and 50 women without breast pathology (H), we found a set of eleven bands (9, 20, 32, 78, 23, 40, 15, 69, 22, 12 and 120) whose recognition by the IgG antibodies in the sera is indicative of BC and/or BBP. Moreover, it was found that this set of bands can differentiate between women with BC and/or BBP from women without breast pathology (H), with a Sensitivity of 2–100% and a Specificity of 56–100%, depending on the number of the BC specific bands which react with the serum’s IgG antibodies [15]. Additionally, most of the sensitivity and specificity values achieved with panels of purified antigens (p53, MUC-1, c-myc gene, BRCA-2, HER-2 …) are below the levels achieved by our method [11,14, 16–20].

Many complex systems in nature, social enterprises and technological achievements can be described in terms of networks. The individual components (nodes) of the network are interrelated through an intricate set of connections (links) that comprise the whole system and define its properties and functions [21–24].

Niels Jerne proposed the Immune Network Theory in the 1970s, stating that the immune system may be viewed as a network of cellular interactions, which at a given moment self-determines its own identity and is self- regulated by idiotype/anti-idiotype interactions [25]. According to this theory, lymphocyte clones are regulated by other lymphocyte clones within a network. In that context, even in the absence of interactions with the external environment, the Immune Network could manifest autonomous activities [26]. Following this perspective, the essence of the immune system is the regulation of lymphocytes by the interaction of their antigen-binding molecules (mainly immunoglobulins), thus implying that the immune system contributes to the integration of an internal environment or molecular ecology [27–30].

In the case of BC, the presence of antibodies against breast tumor antigens may be analyzed as a network in which the connections and the dynamics among the clones can maintain a healthy state, or coexist with a benign pathology or a cancer.

Based on the foregoing, the present article presents an analysis from the perspective of the networks, that is, their composition and geometric structure among the 121 bands (nodes) found in the 1D-II of women with Breast Cancer (BC), with Benign Breast Pathology (BBP) or without breast pathology (H).

The composition and structure of the network in each of the women’s groups, as well as their similarities and differences, might help to identify and understand the mechanisms by which the pathology is triggered, whilst the more connected nodes (hubs) of each network will indicate the antigens and antibodies that are more useful in the design of therapeutic and diagnostic tools, and those that disappear in women with BC or BBP as possibly protective antigens and antibodies.

## Methods

### Grouping of Participants and Sample Sizes

Experimental groups were made up of 50 female patients. Breast cancer patients were recruited, all of which were over 30 years old, with histologically confirmed untreated breast cancer (BC) (94% of the infiltrating canalicular variety, 2% Lobular, 2% Mucinous, 2% Tubular), as well as 50 patients suffering from benign breast pathology (BBP) (fibroadenoma or cystic fibrosis or mastitis) during their first consultation at the Hospital General de México. We also recruited 50 women without breast pathology who were considered healthy (H), older than 22 years of age, without any signs or symptoms of breast pathology (Table 1). All participants were informed of the details of the study (scientific and technical basis, exclusive use of their blood sample for immunodiagnostic of BC, anonymity, confidentiality) and signed a letter of informed consent. The protocol was reviewed and approved by the Committee of Ethical Research at the Hospital General de México.

**Table 1:** Main characteristics of the women cohort.

	BC (n = 50) %	BBP (n = 50) %	H (n = 50) %
**Age group**			
16–19	0	6	0
20–29	6	14	34
30–39	14	28	14
40–49	30	26	28
50–59	26	24	18
60–69	10	0	6
> 70	14	2	0
**Age**			
Xˉ	51.68	40.42	40.57
**SD**	13.62	11.69	12.69
**Age of menarche**			
Xˉ	12.8	12.44	12.47
**SD**	1.52	1.25	1.44
**Number of children**			
Xˉ	2.54	0.93	0.84
**SD**	2.13	1.18	1.31
**Marital status**			
Never married	16	36	58
Married	44	34	32
Free union	18	16	4
Divorced or Separated	6	8	6
Widowed	16	6	0
**Years of education**			
0	6	0	0
1–6	52	26	10
7–12	30	46	16
13–18	12	14	50
>19	0	14	24
**Family history of cancer**			
Yes	40	56	46
No	60	44	54

### Blood Samples

Ten milliliters of venous blood were collected from each participant using sterile and discardable Vacutainer Kits. Blood samples were left to coagulate at room temperature, from which sera were collected after their centrifugation at 1,500 rpm and they were then aliquoted in five sub- samples of equal volume before storage at -80°C.

### Cell Culture and Antigen Preparation

The human breast cancer cell line T47D was obtained from ATCC (Manassas, VA, USA), cultured in phenol-free RPMI 1640 media supplemented with 10% fetal calf serum, 100 U/ml penicillin, 100 mU/ml streptomycin and 250ng/ml amphotericine B. The cells were grown on plastic tissue culture plates (Costar, Cambridge) within atmospheric conditions of 95% humidity and 5% CO_2_ at 37°C. When the cells reached the desired confluence, they were harvested by treatment with Versene (Tris, NaCl, EDTA) and pelleted by centrifugation at 1,500 rpm for 5 minutes. Cell pellets were washed three times with PBS and stored at -80°C.

As a control, the human cell-line MCF10, which was originated from a mammary gland fibrocystic disease, was obtained from ATCC (Manassas, VA, USA). MCF10A cells were grown on Petri dishes with culture medium (2/3 DMEM high glucose, 1/3 F12K) supplemented with 10% FBS, Anti-Anti (Gibco), 2mM glutamine, 1μg/mL Hydrocortisone, 100 mU/mL insulin and 10 ng/mL EGF, and incubated at 37°C. For obtaining protein extracts, once the culture dishes reached a 85–90% confluence, cells were washed with PSB and incubated with Versene for 7 minutes at 37°C. Then, cells were detached from the dish plates washing the surface of the dish plates with a 1 mL pipette tip and once all the cells were detached, the cell suspension was centrifuged at 1150 rpm for 5 minutes and the cell pellet was washed three times with PBS.

Cell pellets destined for 1D-II analysis were lysed for 30 minutes at 4°C in a denaturing lysis buffer composed of 7 M urea, 4% (w/v) CHAPS, 65 mM DTT and Halt protease inhibitor cocktail (PIERCE, 10 μL per 875 μL of lysis buffer), using 40 μl per 10^6^ cells. The lysate was centrifuged at 16,000 g for 10 minutes at 4°C, the supernatant was recuperated and its protein concentration was measured using the commercial Bradford reagent assay (Bio-Rad, Hercules, CA). The different cell lysate batches were pooled, aliquoted and their protein concentrations were similarly measured. Aliquots of the pooled batches were kept at -80°C until further use.

### Uni-dimensional Electrophoresis (1D-Electrophoresis) and Immunoblots of the Protein Extracts from the T47D Cell-Line

100 μg of the pooled protein extract (PE) from the T47D Cell-Line were subjected to electrophoresis using polyacrilamide gels (4–20% TGX Bio-Rad) to separate the proteins by molecular weight, at 80V for about 2 hours. After electrophoresis, the separated proteins were then transferred electrophoretically onto nitrocellulose membranes (HyBond ECL, Amersham Biosciences) using a mini Trans-Blot cell (Bio-Rad; 1h 15 min, 100 V). To confirm the transference of proteins, membranes were reversibly-stained using CPTS (copper phthalocyanine tetrasulphonic acid, ALDRICH) diluted in 12mM HCl, scanned and destained followed by blocking with 5% skimmed Nestlé’s Svelty powdered milk in PBS containing 0.3% Tween 20, pH 7.4 (PBS-T) (16h; 4°C). Each membrane was marked with two pencil lines, one at its upper limit and another one at its lower limit, and then cut vertically into seventeen to eighteen 4 mm wide strips. Each strip was then probed individually with the serum of a different participant diluted 1:300 in 2 mL of fresh blocking solution (5h; room temperature). The same identical serum from a healthy woman (H111) was included in each set of 17–18 strips as a control used to match the problem sera’s electrophoretic runs between the runs of the 17 different membranes. Bound antibodies were detected by incubation with HRP-conjugated second antibody (goat anti-human IgG (H+L); THERMO; 1h, room temperature) diluted 1:2500 in PBS-T. Detection of second antibody binding was performed by incubation with DAB substrate (SIGMA; 0.1 mg/ml, 0.015% peroxide in PBS-T) for 5 minutes at room temperature and the reaction was stopped by rinsing the strips five times with deionized water. Five washes with 2 ml of PBS-T each were performed between each step.

### Image Analysis

The strips of the Immunoblots were left to air-dry overnight, protected from exposure to light and were prepared for scanning. An HP Scanjet G4050 scanner was used to scan all images (reversible-stained membranes and immunoblot strips) at a resolution level of 300 dpi in TIF format. The digitized images of the strips were aligned in Photoshop and then the images were analyzed with Quantity-One software (Bio-Rad) for the detection of bands and calculation of Molecular Weights (MW, kDa). All images were studied with the same settings of brightness, contrast and color to minimize bias.

Banding patterns between strips from different gels and membranes were compared using the control strips (H111) in order to identify the total number of different bands and create a binary database with the presence (1) or absence (0) of each band in each strip.

In order to compare immunoblots obtained with T47D and MCF10 extracts, both extracts were loaded onto 10-well polyacrylamide gels. One gel was stained with Coomassie Blue and the other gel was transferred onto nitrocellulose membranes. Each lane was incubated individually with sera. Three sera were used: 7, 10 and 111. Each serum was probed both for T47D and MCF10 extracts, each in a different lane. The banding patterns obtained with the sera probed with T47D extract were compared to those obtained for the same sera previously; the same was done for the sera probed with MCF10 extract. This allowed the horizontal comparison of all the immunoblots obtained with T47D and MCF10 extracts.

### Network Analysis

In order to establish the presence of a Network among the 121 bands (nodes) of 1D-II and evaluate whether the Networks are different among the groups of women with BC, BBP and H, Pearson correlations were calculated among the bands (nodes) in each group of women, using the Statistical Package for the Social Sciences software (SPSS), as a measure of the statistical validity of the bands’ interconnections by links.

Statistically significance tests were performed by SPSS, which are based on the *t* distribution, then the significant value (p-value) can be obtained through hypothesis testing about rho, the null hypothesis. In this case, the null hypothesis implies that the true correlation is zero and that, therefore, the alternative hypothesis’ correlation is different from zero. Furthermore, the hypothesis testing algorithm suggests that the testing value should be calculated as follows:

$$t=\frac{r\sqrt{n-2}}{\sqrt{1-r^{2}}}$$

Where, *r* is the Pearson´s Correlation Coefficient and *n* is the number of sample observations. Finally, the decisions rule establishes that if |t|>tα2,n−2, where tα2,n−2 is the t distribution value with probability α2 and *n−2* degrees of freedom, thus, the conclusion is the rejection of the null hypothesis, which means that the correlation between two variables is significantly different from zero and it can be considered statistically significant. In terms of probability, the p-values can be obtained by the probability calculation of *t* (*p-value*), based on the t distribution and the decisions rule indicates that if p−value<α2 then the null hypothesis should be rejected [31].

In order to create the Networks, the correlation matrix (*R*) from *k* group was transformed into a Binary matrix (*B*), where *k* = 1,2,3. The transformation indicates that if *r*_*i*,*j*_ (the Pearson’s Correlation Coefficient between variable *i* and variable *j* with *i* ≠ *j*) was considered statistically significant, then, *b*_*i*,*j*_ = 1, if the opposite occurs then *b*_*i*,*j*_ = 0. In this way, the number of Links was given by the number of the ones in *B*, which means the number of significant Pearson Correlations found in the *k* group.

Subsequently, if the Pearson Correlations obtained were statistically significant, they were plotted by Agna software [32]. Agna is a platform-independent application designed for social network analysis, sociometry and sequential analysis. Agna allows the user to create, edit, analyze, store and visualize Networks.

Network Analysis (or Social Network Analysis) is a set of mathematical methods used in social psychology, sociology, ethology, and anthropology [32]. This methodology assumes that the way the members of a group communicate with each other affects some important properties of the group (such as performance, leadership, work satisfaction, etc.). We propose that equivalent actors (*i*.*e*., tumor cells and clones of antibody producing cells) and equivalent immunological events (*i*.*e*., protein synthesis, expression and secretion, cellular differentiation, reproduction or death), occur in immunological networks and affect the performance of the tumor cells (*i*.*e*., tissue invasion, metastasis) and of the immune system of the patient with BC in successfully or disastrously dealing with the tumor cells.

Sequential analysis deals with chains of behavior by way of recording the behavior of an animal under specific personal and circumstantial conditions and, then, by dividing the complete set of behavior types into basic sequential units, or links, to make with them a single filed sequential chain of nodes [32].

Here we studied the differences between the 121 bands of the 1D-II of the 50 women with Breast Cancer (BC), those with Benign Breast Pathology (BBP) or without breast pathology (H), and we postulate that the clones of IgG-producing cells are the equivalent components of the immune Network, and adopt the equivalent structures and behavior found in the social networks.

Statistical differences in the number of nodes and links between groups were calculated with the Kruskall-Wallis test. The differences in the intensity of the networks’ Connectivity among the groups of women were also analyzed. In order to compare the connection Intensity, it is necessary to define two measures. First, the number of significant correlations in *k* group is denoted by *N*_*k*_. Then, the expected value of all the significant correlations in *k* group is calculated by the following formula:

$$M_{k}=\frac{\sum_{i=1}^{m}r_{i,k}}{m}$$

Where *r*_*i*,*k*_ represents the significant correlation *i* of the group *k*. Finally, the connection Intensity (*I*_*i*_) can be calculated as follows:

$$I_{k}=M_{k}*N_{k}$$

As can be inferred from the equation above, *I*_*k*_ is a measure which indicates the expected value of Links in group *k*, based on the Pearson´s Correlations between nodes.

Also, disconnected Nodes were identified using the follow algorithm:

$$\begin{array}{c} \mathrm{if}\;\left(a==1\&\&b==0\right)\;\mathrm{output}=2\\ \mathrm{else if}\;\left(a==0\&\&b==1\right)\;\mathrm{output}=1\\ \mathrm{else if}\;\left(a==1\&\&b==1\right)\;\mathrm{output}=0\\ \mathrm{else}\;\left(a==0\&\&b==0\right)\;\mathrm{output}=0 \end{array}$$

by applying it as a logical formula in the Excel program addressing the corresponding Excel worksheets. Values (1,0) assigned to “a” and “b” correspond to link presence (1) or link absence (0) between the matrix compared (H vs BBP; H vs BC and BBP vs BC). All matrices were studies to identify output = 2 which point a lost connection, and then a table of disconnections by nodes was constructed.

The network analysis for both T47D and MCF10 were carried out in a similar way.

## Results

### The Networks’ Attributes and Comparisons among the Different Groups of Women

**Fig 1:**
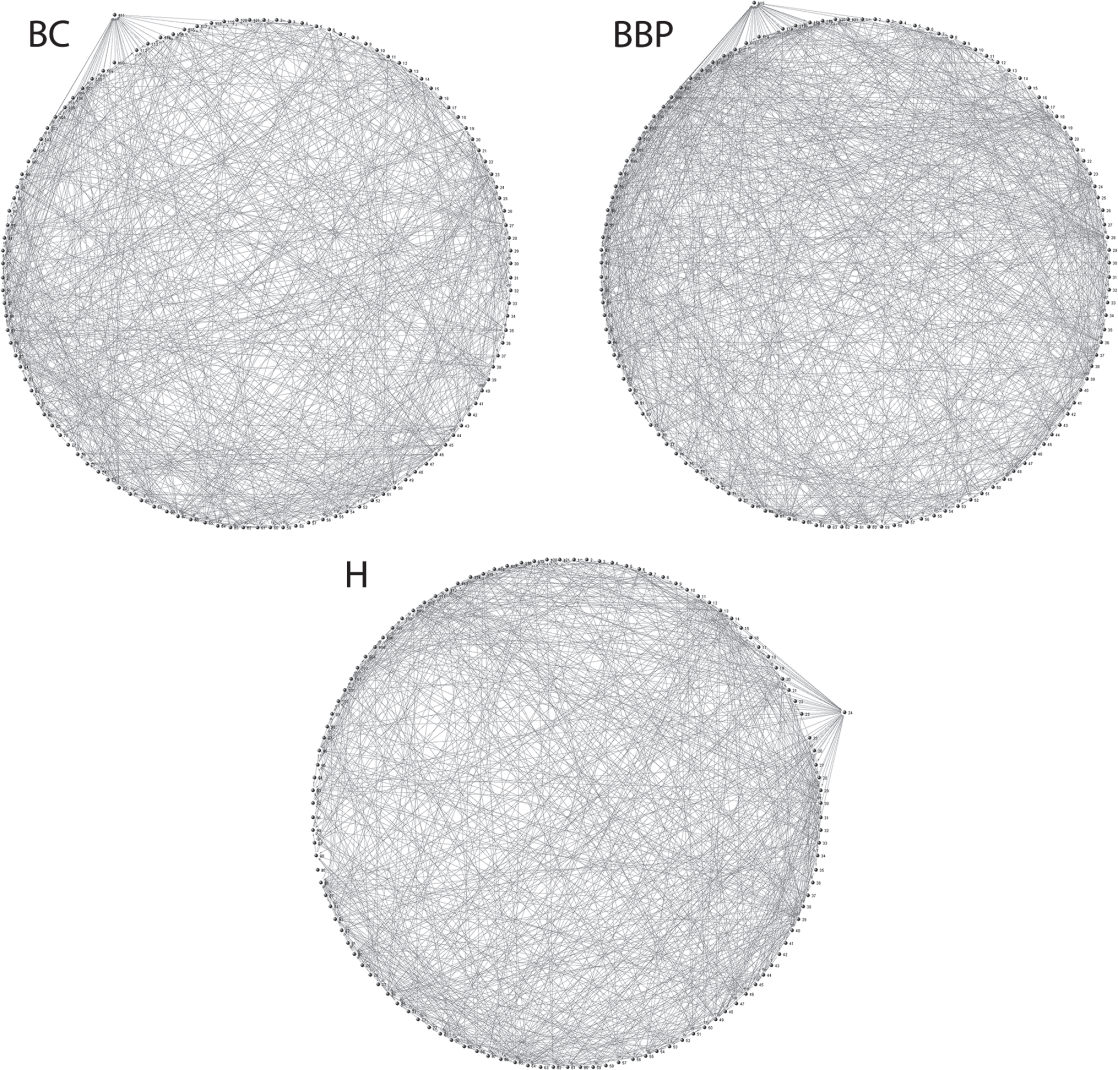
Network visualization.

Table 2 shows the networks’ attributes in each group of the participating women, while Fig. 1 exhibits the networks’ visual features. The network with the highest number of links is that of BBP (1932), followed by H (1642) and finally by BC (1610).

**Table 2:** Network properties per group.

	H	BBP	BC
Number of Nodes	121	121	121
Number of Links	1642	1932	1610
Diameter	7	6	7
Density	0.06	0.07	0.06
Node with more sociometric degree	24	115	111
Minimum Node number	2	2	2
Maximum Node number	39	45	32
Node number means	13.57	15.93	13.28
Standard deviation	7.23	9.56	6.68
Variance	52.26	91.35	44.59

Number of Nodes: maximum number of shared bands found in the 1D-II of women with Breast Cancer (BC), with Benign Breast Pathology (BBP) or without breast pathology (H).

Number of Links: maximun number of connections between the Nodes of each of the compared groups (BC, BBP or H).

Diameter: maximum shortest path between two Nodes measured by the number of routed Links.

Density: proportion of Links in a Network relative to the total number of possible Links.

Node with more sociometric degree: number of the Node (per group) with more connections.

Minimum Node number: minimum number of connected Nodes in a Network.

Maximum Node number: maximum number of connected Nodes in a Network

Despite their numerical differences in connectivity, these numbers were not statistically different from each other (P = 0.207). However, the most connected node (hub) varies among networks: for example, the hub in the H group is node 24 (39 links), for the BBP group it is node 115 (45 links), and node 111 for the BC group (32 links), but there are many other differences in this respect among the networks of the different groups of women (Table 2).

**Fig 2:**
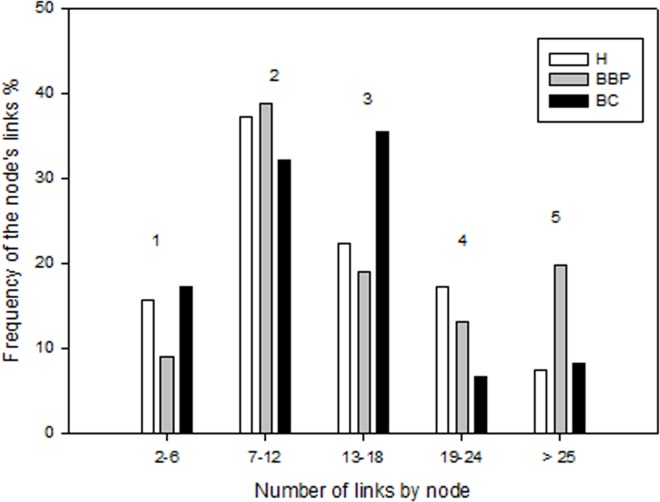
Frequency of the number of Links per Node per group. The Frequency (%) was grouped in five classes according to the number of links in the nodes: 1) 2 to 6 links, 2) 7 to 12 links, 3) 13 to 18 links, 4) 19 to 24 links and 5) 25 and more links. The nodes with more than 19 links were considered as hubs. By this definition, less than the 10% of the nodes in BC should be considered as hubs. Also BC group is the one that present the lower frequency of hubs, followed by H and BBP group.

It is noteworthy that the analysis of the frequency (%) in the number of links in each node shows some differences among the groups of women: particularly striking is the decrease in the number of nodes having 19 to 24 links, which is also more pronounced in the groups of women having some pathology: from 17.4% in H, to 13.12% in BBP, and to 6.6% in BC (Fig. 2).

On the other hand, if one takes into account not only the number of links in each node (N, the number of statistically significant correlations), but also their magnitude (M, the average value of their correlation coefficients of the significant correlations in each node), as well as their Intensity (I = NxM), and assigns an intensity ranking order of the most connected nodes (hubs), it can be seen that the network of H women shares 5 of the 10 most connected nodes (hubs 24, 112, 115, 106, and 39) with the BBP women’s network, while it only shares a single hub (hub 55) with that of the BC group (Table 3). A similar pattern was observed in the MCF10 networks (S1 Table).

**Table 3:** Classification of Immunological Bands according to the Number (N) of significant correlations (P<0.05), the Average Magnitude (M) of the correlations, the Connection Intensity (I = NxM) and its Intensity Ranking Order (R).

Node	BC				BBP				H			
	N	M	I	R	N	M	I	R	N	M	I	R
**Band 24**	14	0.06	0.85	25	42	0.24	10.15	**1**	39	0.25	9.87	**1**
**Band 55**	17	0.27	4.59	**1**	13	0.13	1.65	18	22	0.22	4.89	**2**
**Band 112**	11	0.05	0.56	32	29	0.15	4.27	**4**	30	0.13	4.03	**3**
**Band 115**	14	0.07	0.97	23	45	0.08	3.6	**5**	25	0.13	3.16	**4**
**Band 106**	12	0.08	0.91	24	39	0.09	3.4	**8**	30	0.1	3.02	**5**
**Band 109**	16	0	-0.01	74	23	0.05	1.07	26	24	0.11	2.55	**6**
**Band 7**	9	0	-0.03	78	8	0.02	0.18	83	15	0.13	1.88	**7**
**Band 119**	2	0.01	0.01	67	7	0.06	0.39	69	16	0.11	1.8	**8**
**Band 39**	17	0.02	0.36	37	35	0.09	3.12	**9**	30	0.06	1.75	**9**
**Band 86**	12	0.02	0.27	41	18	0.05	0.87	34	24	0.06	1.47	**10**
**Band 14**	26	0.04	1.12	21	16	0.07	1.1	25	20	0.07	1.46	11
**Band 113**	6	0.02	0.12	56	9	0.16	1.41	20	9	0.16	1.41	12
**Band 5**	19	0.03	0.62	31	16	0.06	1.04	27	16	0.09	1.37	13
**Band 38**	11	-0.01	-0.16	97	14	0.04	0.51	60	25	0.05	1.34	14
**Band 29**	9	0	0.02	65	21	0.08	1.73	16	26	0.05	1.33	15
**Band 44**	17	0.14	2.4	**9**	9	0.07	0.66	52	21	0.06	1.31	16
**Band 10**	3	-0.03	-0.1	94	9	0.14	1.28	21	9	0.14	1.28	17
**Band 40**	12	-0.01	-0.09	93	7	-0.02	-0.17	109	21	0.06	1.28	18
**Band 15**	15	-0.05	-0.79	120	6	-0.01	-0.08	105	9	0.14	1.22	19
**Band 116**	17	0.07	1.26	19	19	0.05	0.87	36	9	0.13	1.21	20
**Band 28**	17	0.14	2.45	**3**	33	0.02	0.58	56	22	0.05	1.19	21
**Band 30**	17	0.15	2.59	**2**	15	0.03	0.44	64	24	0.05	1.18	22
**Band 46**	23	0	-0.01	73	11	0.01	0.08	91	22	0.05	1.11	23
**Band 120**	6	0.02	0.12	55	24	0.03	0.84	37	15	0.07	1.09	24
**Band 75**	17	0.04	0.65	30	9	0.07	0.66	53	21	0.05	1.06	25
**Band 80**	17	0.14	2.43	**5**	7	0.01	0.06	92	21	0.05	0.97	26
**Band 96**	7	0	-0.03	79	32	0.08	2.58	13	23	0.04	0.96	27
**Band 19**	16	-0.01	-0.12	95	26	0.07	1.85	14	17	0.06	0.96	28
**Band 103**	27	0.05	1.22	20	35	0.1	3.48	**6**	14	0.07	0.92	29
**Band 2**	4	0	-0.01	72	9	0.53	4.8	**2**	6	0.15	0.92	30
**Band 9**	15	-0.03	-0.49	115	7	0.01	0.06	93	9	0.1	0.88	31
**Band 17**	6	0	0	68	20	0.04	0.73	42	14	0.06	0.82	32
**Band 100**	9	0.02	0.19	46	24	0.04	0.93	32	12	0.07	0.81	33
**Band 65**	16	0.04	0.69	29	9	-0.01	-0.05	102	26	0.03	0.81	34
**Band 67**	27	0.01	0.37	36	12	0.04	0.53	58	16	0.05	0.78	35
**Band 34**	6	0	0	69	14	0.03	0.44	63	18	0.04	0.77	36
**Band 71**	17	0.14	2.4	**8**	9	0.08	0.68	48	23	0.03	0.75	37
**Band 89**	17	0.13	2.15	13	28	0.01	0.41	68	9	0.08	0.73	38
**Band 51**	17	0.14	2.4	**7**	11	0.01	0.08	90	9	0.08	0.71	39
**Band 118**	8	0.02	0.13	52	24	-0.08	-1.82	121	14	0.05	0.7	40
**Band 47**	9	0.03	0.23	44	8	0.01	0.11	87	9	0.08	0.7	41
**Band 93**	26	0.03	0.75	27	39	0.07	2.92	**10**	15	0.05	0.69	42
**Band 31**	9	-0.02	-0.22	103	9	0.07	0.66	54	16	0.04	0.69	43
**Band 50**	25	-0.02	-0.38	114	7	0.01	0.1	88	16	0.03	0.55	44
**Band 13**	7	0.01	0.06	61	11	-0.03	-0.34	112	24	0.02	0.54	45
**Band 102**	10	-0.05	-0.49	116	8	-0.04	-0.36	113	22	0.02	0.51	46
**Band 114**	8	0.02	0.13	53	9	0.11	0.96	30	13	0.04	0.48	47
**Band 20**	13	0.01	0.1	57	7	0.06	0.42	66	17	0.03	0.45	48
**Band 53**	8	0.01	0.06	62	19	0.04	0.69	46	14	0.03	0.45	49
**Band 85**	30	0.05	1.53	18	33	0.08	2.77	12	9	0.05	0.43	50
**Band 83**	5	-0.01	-0.06	87	9	0	-0.04	101	17	0.02	0.39	51
**Band 12**	11	0.01	0.08	58	11	0.05	0.53	59	21	0.02	0.39	52
**Band 27**	22	-0.01	-0.26	106	18	0.01	0.23	80	8	0.05	0.38	53
**Band 74**	14	0.03	0.47	34	16	0.04	0.69	47	11	0.03	0.35	54
**Band 117**	8	-0.09	-0.73	119	7	-0.06	-0.39	114	9	0.04	0.35	55
**Band 68**	7	0	0.03	63	18	0.02	0.42	67	12	0.03	0.35	56
**Band 4**	4	-0.01	-0.06	86	7	0.1	0.73	41	9	0.04	0.35	57
**Band 78**	8	0	0	71	27	0.07	1.8	15	8	0.04	0.33	58
**Band 3**	10	0.07	0.75	28	11	0.03	0.28	73	12	0.02	0.25	59
**Band 121**	4	0.02	0.08	60	9	0.08	0.68	51	9	0.02	0.22	60
**Band 52**	17	0.14	2.44	**4**	9	0.08	0.68	49	12	0.02	0.22	61
**Band 11**	17	0.12	2.09	15	5	0.03	0.17	84	20	0.01	0.22	62
**Band 92**	16	0.01	0.14	51	15	0.02	0.24	78	10	0.02	0.22	63
**Band 48**	4	-0.01	-0.02	77	20	0.06	1.15	24	12	0.02	0.22	64
**Band 18**	4	-0.01	-0.06	85	23	0.04	0.87	35	8	0.03	0.21	65
**Band 21**	17	0.13	2.13	14	17	0.01	0.19	81	15	0.01	0.19	66
**Band 33**	7	0	-0.02	76	13	0.03	0.44	62	15	0.01	0.18	67
**Band 57**	8	0	-0.04	81	22	0.05	1.16	23	8	0.02	0.17	68
**Band 66**	12	-0.02	-0.21	100	27	0.06	1.62	19	17	0.01	0.17	69
**Band 90**	18	0.01	0.12	54	19	0.04	0.83	38	13	0.01	0.16	70
**Band 61**	12	0.01	0.16	49	5	0	-0.02	99	19	0.01	0.16	71
**Band 26**	9	-0.03	-0.3	109	6	0.02	0.13	85	18	0.01	0.15	72
**Band 82**	17	0.14	2.35	**10**	25	0.03	0.73	43	12	0.01	0.15	73
**Band 1**	17	0.14	2.41	**6**	9	0.08	0.68	50	8	0.02	0.14	74
**Band 58**	4	0	-0.01	75	3	0.01	0.03	95	7	0.02	0.12	75
**Band 107**	29	0.03	0.76	26	30	0.11	3.43	**7**	5	0.02	0.1	76
**Band 35**	21	0.01	0.26	42	15	0.05	0.77	39	4	0.02	0.09	77
**Band 79**	12	-0.02	-0.26	105	8	0.01	0.11	86	4	0.02	0.07	78
**Band 69**	14	-0.02	-0.35	112	8	-0.04	-0.29	111	10	0.01	0.07	79
**Band 8**	6	-0.04	-0.21	102	36	0.05	1.72	17	4	0.02	0.07	80
**Band 87**	27	0.02	0.44	35	14	0.06	0.9	33	2	0.02	0.04	81
**Band 42**	4	0.02	0.08	59	28	0.04	1.03	28	5	0.01	0.04	82
**Band 60**	14	0.02	0.23	43	22	0.02	0.36	70	4	0.01	0.03	83
**Band 91**	12	-0.02	-0.24	104	9	0.03	0.23	79	3	0.01	0.02	84
**Band 73**	9	-0.02	-0.17	98	8	-0.01	-0.08	104	7	0	0.02	85
**Band 63**	14	0.02	0.23	45	2	-0.01	-0.02	100	3	0	0.01	86
**Band 16**	6	-0.03	-0.15	96	13	0.06	0.72	45	13	0	0.01	87
**Band 23**	31	-0.01	-0.37	113	21	-0.08	-1.67	120	4	0	-0.02	88
**Band 43**	16	0.01	0.18	47	7	-0.02	-0.15	108	7	0	-0.02	89
**Band 22**	6	-0.01	-0.04	84	7	-0.03	-0.21	110	7	0	-0.03	90
**Band 101**	3	-0.02	-0.07	89	24	0.12	2.9	11	7	-0.01	-0.04	91
**Band 41**	11	0	0	70	5	0.05	0.25	77	5	-0.01	-0.06	92
**Band 94**	18	-0.04	-0.73	118	17	0.02	0.27	75	5	-0.01	-0.07	93
**Band 56**	15	-0.01	-0.08	91	4	-0.02	-0.08	106	11	-0.01	-0.1	94
**Band 97**	6	-0.06	-0.34	111	25	0.02	0.6	55	3	-0.03	-0.1	95
**Band 88**	22	-0.05	-1.18	121	8	-0.01	-0.07	103	9	-0.01	-0.11	96
**Band 54**	16	-0.02	-0.28	108	12	0.02	0.3	72	6	-0.02	-0.12	97
**Band 81**	17	0.02	0.28	40	4	0.01	0.04	94	6	-0.02	-0.13	98
**Band 45**	20	-0.01	-0.26	107	10	0.07	0.74	40	10	-0.01	-0.13	99
**Band 84**	17	0.13	2.25	12	5	0.02	0.09	89	9	-0.02	-0.15	100
**Band 72**	20	0.01	0.17	48	23	0.04	0.96	31	6	-0.03	-0.16	101
**Band 77**	7	-0.01	-0.06	88	8	0.03	0.28	74	6	-0.03	-0.17	102
**Band 32**	12	0.01	0.15	50	6	0	0.02	97	12	-0.02	-0.19	103
**Band 6**	16	-0.01	-0.19	99	12	0.04	0.43	65	8	-0.03	-0.2	104
**Band 25**	17	0.14	2.3	11	8	-0.01	-0.1	107	9	-0.02	-0.22	105
**Band 111**	32	0.03	1.02	22	34	0.13	4.57	**3**	12	-0.02	-0.23	106
**Band 76**	15	0	0.02	64	10	0.03	0.26	76	25	-0.01	-0.28	107
**Band 37**	7	-0.01	-0.04	83	19	0.02	0.46	61	11	-0.03	-0.29	108
**Band 105**	6	-0.03	-0.21	101	27	0.04	1.02	29	4	-0.07	-0.29	109
**Band 98**	14	0.04	0.56	33	11	0	0.01	98	12	-0.02	-0.29	110
**Band 36**	9	0	-0.04	82	27	-0.04	-1.15	119	9	-0.03	-0.3	111
**Band 104**	11	0.03	0.32	39	9	-0.07	-0.66	118	17	-0.02	-0.3	112
**Band 59**	19	0.02	0.34	38	11	0.02	0.18	82	14	-0.02	-0.34	113
**Band 95**	17	0.12	2.01	16	14	-0.05	-0.64	117	11	-0.04	-0.42	114
**Band 108**	9	0	-0.03	80	12	0.05	0.55	57	9	-0.05	-0.44	115
**Band 70**	8	0	0.01	66	16	0.05	0.73	44	13	-0.04	-0.51	116
**Band 99**	17	0.1	1.77	17	16	0.02	0.34	71	9	-0.07	-0.6	117
**Band 49**	8	-0.01	-0.08	92	17	-0.03	-0.46	115	21	-0.04	-0.81	118
**Band 62**	15	-0.04	-0.54	117	26	-0.02	-0.55	116	16	-0.06	-0.9	119
**Band 64**	16	-0.02	-0.34	110	14	0	0.03	96	21	-0.06	-1.21	120
**Band 110**	4	-0.02	-0.07	90	25	0.05	1.22	22	24	-0.05	-1.24	121
**Totals**	1610	2.70	50.30		1932	4.83	95.52		1642	3.63	67.32	

Bolded and underlined are the most connected nodes ranked from 1 to 10.

Also, unlike the analysis in the total number of links, when comparing the overall I (ΣI) in each of the networks, there are statistically significant differences (P = 0.001) among the networks of the women’s groups (Xˉ = 0.5568595 in H, Xˉ = 0.78975207 in BBP and Xˉ = 0.41578512 in BC) (Table 3).

As previously noted, the comparisons among networks show that there are significant differences in their connection intensities. Table 4 shows the number of links that are missing in each node and the nodes with which each node lost its links.

**Table 4:** Network Disconnections.

H vs BC			H vs BBP			BBP vs BC		
Node	Number of Disconnections	Disconnected Nodes	Node	Number of Disconnections	Disconnected Nodes	Node	Number of Disconnections	Disconnected Nodes
24	27	27, 29, 39,41, 46, 48, 58, 59, 61, 64, 65, 68, 69, 73, 76, 92, 93, 96, 98, 102, 106, 109, 110, 112, 115, 120, 121	11	19	13, 15, 20, 26, 28, 30, 34, 38, 45, 50, 55, 62, 67, 71, 75, 80, 86, 104, 111	24	33	27, 28, 34, 35, 36, 39, 42, 48, 57, 60, 64, 65, 69, 72, 76, 85, 86, 90, 92, 93, 95, 96, 100, 103, 106, 107, 109, 110, 111, 112, 115, 118, 119
13	22	27, 28, 30, 32, 36, 38, 40, 44, 49, 50, 55, 56, 67, 70, 75, 76, 80, 86, 108, 110, 111, 112	13	16	27, 28, 30, 32, 36, 38, 40, 44, 49, 50, 71, 80, 86, 110, 111, 112	8	31	14, 17, 23, 34, 35, 36, 39, 42, 48, 49, 53, 54, 57, 60, 62, 66, 69, 70, 87, 90, 93, 96, 100, 101, 103, 106, 107, 111, 112, 115, 120
12	21	14, 21, 23, 29, 33, 38,41,57, 64, 66, 76, 84, 85, 96, 109, 110, 112, 115, 119	26	15	28, 34, 38, 40, 44, 50, 52, 55, 61, 70, 71, 75, 80, 82, 86	28	22	37, 47, 53, 60, 62, 72, 85, 93, 94, 97, 100, 101, 103, 105, 106, 107, 108, 109, 110, 115, 117, 118
30	16	33,35,40, 42, 50, 51, 61, 67, 68, 78, 95, 98, 111, 112, 114, 117	28	15	30, 34, 38, 40, 44, 45, 50, 65, 67, 71, 75, 80, 86, 104, 111	18	20	28, 60, 62, 77, 82, 85, 89, 92, 93, 96, 97, 101, 102, 103, 106, 109, 110, 115, 116, 118
29	16	30, 31, 35, 46, 48, 64, 68, 70, 74, 94, 106, 109, 110, 112, 119, 120	20	14	28, 40, 43, 44, 55, 62, 65, 69, 71, 72, 75, 80, 86, 98,	36	20	39, 43, 46, 49, 60, 64, 66, 69, 70, 86, 96, 102, 103, 104, 106, 107, 111, 112, 115, 120
11	15	13, 20, 26, 34, 38, 45, 50, 61, 62, 65, 67, 75, 86, 105, 111	24	14	29, 41, 46, 49, 58, 59, 61, 68, 73, 83, 98, 102, 104, 121	42	18	49, 57, 62, 66, 78, 85, 86, 90, 93, 96, 101, 103, 106, 107, 109, 111, 112, 115
39	14	53, 64, 66, 74, 76, 83, 93, 96, 99, 100, 102, 104, 120, 121	46	14	74, 83, 86, 88, 92, 93, 98, 102, 106, 109, 110, 112, 119, 121	19	17	24, 39, 57, 72, 76, 78, 85, 93, 96, 101, 103, 106, 107, 111, 112, 115, 120
38	14	40, 44, 45, 49, 55, 59, 65, 67, 75, 80, 93, 110, 112, 118	16	13	52, 62, 71, 75, 77, 80, 82, 84, 88, 100, 105, 109, 118	17	16	35, 39, 42, 53, 74, 81, 86, 87, 92, 93, 94, 97, 103, 106, 110, 115
26	14	28, 30, 34, 38, 44, 50, 52, 55, 61, 70, 75, 80, 82, 86	30	13	31, 40, 44, 48, 55, 65, 66, 76, 93, 96, 109, 112, 115	21	14	30, 33, 36, 38, 39, 46, 85, 103, 105, 106, 110, 115, 117, 118
19	14	24, 29, 39, 53, 57, 68, 78, 79, 85, 93, 101, 106, 107, 120	38	13	40, 44, 45, 55, 65, 67, 71, 75, 76, 86, 93, 112, 118	60	14	85, 89, 93, 94, 96, 100, 103, 105, 106, 109, 110, 112, 115, 118
20	13	28, 43, 44, 55, 62, 65, 69, 72, 75, 80, 86, 98, 114	40	13	44, 45, 50, 55, 61, 65, 67, 71, 75, 80, 86, 104, 111	16	13	18, 30, 33, 37, 38, 41, 55, 67, 83, 96, 98, 99, 106,
14	13	24, 29, 31, 39, 46, 49, 70, 73, 83, 102, 104, 106, 121	5	12	7, 14, 15, 17, 18, 65, 70, 73, 96, 102, 120, 121	37	13	55, 67, 77, 79, 82, 92, 94, 99, 105, 108, 110, 116, 120
5	13	7, 14, 18, 19, 24, 39, 65, 70, 73, 78, 102, 106, 121	14	12	23, 31, 46, 49, 68, 73, 74, 75, 83, 102, 106, 121	57	13	60, 68, 85, 90, 92, 93, 100, 103, 106, 111, 112, 115, 118
40	12	45, 50, 55, 61, 62, 65, 67, 75, 80, 86, 104, 111,	34	12	40, 44, 52, 55, 61, 67, 71, 75, 80, 82, 95, 104	62	13	72, 89, 93, 95, 96, 97, 100, 103, 105, 106, 108, 109, 115
28	12	38, 40, 45, 50, 61, 62, 65, 67, 75, 86, 104, 111	39	12	53, 64, 74, 76, 83, 99, 100, 102, 104, 109, 119, 121	5	12	8, 19, 24, 27, 34, 39, 48, 57, 78, 90, 106, 115
17	12	19, 24, 29, 31, 39, 68, 76, 97, 106, 112, 115, 119	3	11	14, 31, 46, 53, 57, 79, 83, 90, 93, 106, 114	29	12	36, 45, 48, 64, 89, 96, 103, 105, 107, 112, 119, 120
16	12	21, 52, 62, 71, 75, 80, 82, 84, 100, 105, 109, 118	12	11	14, 23, 57, 64, 66, 76, 85, 96, 112, 115, 119	66	12	72, 78, 85, 88, 90, 93, 98, 101, 107, 111, 112, 115
3	12	14, 31, 46, 53,57, 74, 79, 83, 90, 93, 106, 114	32	11	38, 49, 64, 72, 73, 76, 96, 98, 109, 110, 112	68	12	93, 94, 95, 96, 97, 10, 102, 103, 106, 115, 116, 118
32	11	38, 49, 64, 72, 73, 76, 96, 98, 109, 110, 112,	44	11	45, 50, 55, 61, 62, 65, 67, 80, 86, 104, 111	78	12	85, 86, 90, 93, 96, 101, 102, 103, 111, 112, 115, 120
21	11	33, 36, 76, 77, 105, 109, 110, 112, 115, 118, 119	7	10	14, 31, 46, 49, 65, 90, 101, 102, 106, 107	3	11	4, 11, 12, 20, 26, 30, 33, 38, 59, 74, 84
7	11	14, 19, 24, 31, 46, 49, 90, 101, 102, 106, 107	29	10	30, 46, 66, 70, 74, 83, 94, 106, 109, 110	13	11	37, 55, 56, 63, 67, 72, 76, 79, 90, 100, 108
96	10	99, 102, 106, 108, 109, 110, 112, 115, 119, 120	65	10	69, 71, 86, 88, 90, 98, 103, 110, 114, 118	14	11	23, 28, 29, 39, 70, 78, 89, 90, 101, 104, 120
44	10	45, 50, 61, 62, 65, 67, 75, 86, 104, 111	33	9	38, 57, 59, 76, 84, 85, 109, 112, 118	39	11	42, 48, 57, 66, 86, 90, 93, 96, 101, 107, 120
37	10	53, 60, 64, 76, 91, 95, 97, 104, 106, 108	37	9	53, 60, 64, 76, 91, 95, 97, 104, 106	48	11	53, 58, 62, 85, 87, 96, 103, 106, 111, 112, 115
34	10	40, 44, 52, 55, 75, 80, 82, 86, 95, 104	50	9	53, 55, 66, 69, 76, 86, 95, 100, 114	82	11	92, 93, 97, 100, 101, 105, 106, 107, 108, 110, 118
31	10	39, 48, 49, 64, 66, 69, 83, 106, 112, 115	55	9	61, 62, 65, 71, 75, 80, 86, 104, 111	89	11	93, 97, 100, 101, 105, 106, 109, 110, 115, 118, 119
49	9	59, 64, 96, 101, 108, 109, 110, 112, 115	61	9	62, 71, 75, 80, 81, 82, 86, 92,104	6	10	30, 33, 38, 39, 66, 68, 83, 104, 112
46	9	83, 86, 88, 93, 102, 109, 110, 119, 121	64	9	66, 76, 83, 84, 88, 96, 106, 112, 115	27	10	36, 42, 62, 78, 85, 93, 101, 106, 112, 115
33	9	57, 59, 64, 76, 84, 85, 109, 110, 112	2	8	7, 21, 29, 33, 35, 67, 84, 101	34	10	36, 39, 66, 85, 86, 93, 106, 112, 115, 120
64	8	76, 83, 84, 88, 96, 106, 112, 115	4	8	9, 10, 47, 51, 89, 113, 116, 117,	53	10	57, 62, 65, 69, 70, 78, 89, 90, 106, 115
55	8	61, 62, 65, 67, 75, 86, 104, 111	17	8	19, 29, 31, 68, 76, 109, 112, 119	96	10	100, 103, 104, 106, 107, 109, 111, 112, 115, 119
50	8	55, 65, 69, 76, 86, 95, 100, 114	25	8	34, 37, 52, 53, 61, 75, 82, 104	2	9	10, 31, 44, 52, 71, 75, 113, 114, 121
6	8	26, 29, 39, 64, 66, 68, 70, 106	31	8	48, 49, 64, 66, 69, 83, 106, 115	7	9	19, 23, 24, 39, 66, 73, 88, 93, 118
4	8	9, 10, 15, 47, 51 89, 113, 116	76	8	86, 95, 109, 110, 112, 115, 119, 120	12	9	21, 30, 33, 38, 41, 59, 84, 109, 110
1	8	2, 7, 18, 24, 34, 48, 67, 102	9	7	10, 47, 51, 89, 113, 116, 117	30	8	38, 46, 49, 59, 64, 83, 92, 110
102	7	106, 110, 111, 112, 115, 119, 121	49	7	59, 96, 101, 108, 109, 110, 112	51	8	53, 68, 72, 94, 97, 105, 106, 117
76	7	86, 95, 96, 109, 110, 119, 120	62	7	71, 75, 80, 82, 86, 119, 120	54	8	76, 79, 82, 101, 107, 117, 118, 120
65	7	69, 86, 88, 90, 103, 110, 118	102	7	106, 109, 111, 112, 119, 121	70	8	78, 82, 89, 99, 101, 107, 118, 120
53	7	54, 56, 70, 95, 100, 106, 111	1	6	7, 18, 34, 48, 67, 102	85	8	89, 95, 96, 100, 105, 110, 112, 118
10	7	15, 47, 51, 89, 113, 116, 117	10	6	15, 47, 51, 89, 116, 117	93	8	95, 96, 97, 100, 105, 106, 112, 118
9	7	10, 15, 47, 51, 113, 116, 117	21	6	76, 77, 84, 109, 112, 119	105	8	106, 108, 109, 110, 115, 116, 118, 119
58	6	59, 66, 74, 90, 92, 102,	22	6	26, 36, 42, 59, 65, 94	10	7	44, 52, 71, 75, 113, 114, 121
56	6	76, 82, 90, 96, 100, 103,	52	6	56, 61, 62, 80, 82, 86	23	7	28, 39, 42, 54, 70, 98, 101
36	6	46, 56, 90, 94, 111, 112	53	6	54, 56, 70, 95, 100, 111	31	7	39, 45, 76, 93, 96, 111, 112
25	6	34, 37, 53, 61, 75, 104	56	6	76, 82, 90, 96, 100, 103	35	7	39, 42, 48, 89, 96, 106, 109
22	6	26, 36, 42, 59, 65, 94	58	6	59, 66, 74, 90, 92, 102	38	7	46, 49, 59, 64, 83, 92, 110
18	6	24, 27, 46, 63, 73, 92	59	6	60, 65, 70, 98, 103, 110	47	7	50, 56, 74, 86, 89, 95, 99
15	6	47, 51, 89, 113, 116, 117	6	5	26, 29, 64, 70, 106	94	7	97, 100, 103, 105, 110, 116, 117
109	5	110, 112, 115, 118, 119	15	5	47, 51, 113, 116, 117	97	7	99, 100, 101, 108, 115, 116, 118
99	5	100, 104, 106, 108, 109	18	5	23, 27, 46, 63, 73	1	6	2, 10, 75, 113, 114, 121
95	5	96, 100, 104, 106, 108	43	5	65, 98, 109, 114, 118	4	6	30, 33, 38, 66, 74, 98
83	5	102, 111, 112, 115, 121	48	5	54, 64, 66, 83, 101	15	6	21, 25, 80, 89, 97, 98
70	5	81,93,98, 106, 107,	67	5	71, 75, 80, 81, 86	22	6	55, 80, 88, 89, 99, 105
68	5	83, 90, 93, 112, 115	68	5	71, 83, 88, 90,112	25	6	32, 60, 62, 79, 87, 88
66	5	76, 83, 90, 106, 115	71	5	72, 80, 82, 86, 118	33	6	46, 49, 64, 83, 92, 110
62	5	75, 80, 82, 119, 120	83	5	102, 111, 112, 115, 121	40	6	48, 49, 51, 54, 62, 97
59	5	60, 65, 70, 98, 110	99	5	100, 104, 106, 108, 1092	86	6	93, 96, 103, 106, 112, 115
52	5	56, 61, 62, 75, 86	19	4	29, 53, 68, 79	95	6	96, 97, 100, 106, 15, 118
48	5	54, 64, 66, 83, 115	47	4	51, 113, 116, 117	100	6	105, 106, 109, 115, 116, 118
47	5	51, 89, 113, 116, 117	66	4	76, 83, 102, 106	101	6	105, 107, 112, 115, 116, 120
2	5	7, 33, 67, 84, 101	69	4	88, 98, 103, 114	26	5	27, 74, 98, 102, 120
100	4	106, 112, 115, 118	70	4	81, 93, 98, 106	43	5	50, 64, 81, 90, 91
90	4	99, 102, 103, 110	75	4	80, 81, 82, 86	49	5	62, 64, 107, 111, 115
78	4	85, 103, 111, 120	92	4	102, 111, 112, 121	55	5	67, 77, 94, 105, 110
75	4	80, 81, 82, 86	96	4	99, 102, 108, 120	74	5	85, 91, 108, 109, 115
74	4	75, 91, 93, 119	23	3	43, 84, 114	9	4	20, 42, 83, 86
67	4	75, 80, 82, 86	27	3	30, 39, 45	44	4	75, 113, 114, 121
61	4	75, 80, 81, 82	35	3	46, 74, 87	45	4	48, 102, 104, 107
57	4	78, 85, 114, 120	36	3	56, 90, 94	52	4	75, 113, 114, 121
54	4	77, 79, 110, 118	41	3	64, 76, 96	59	4	68, 83, 84, 92
45	4	49, 55, 87, 104	42	3	45, 94, 120	67	4	77, 82, 99, 116
42	4	45, 94, 112, 120	45	3	49, 55, 87	71	4	75, 113, 114, 121
27	4	39, 45, 93, 106	57	3	78, 114, 120	72	4	73, 78, 87, 115
112	3	118, 119, 120	72	3	86, 98, 101	73	4	76, 102, 107, 111
106	3	108, 118, 119	74	3	93,114, 119	77	4	88, 91, 99, 108
92	3	110, 112, 121	77	3	100, 105, 118	106	4	109, 110, 111, 118
72	3	86, 98, 101	80	3	81, 82, 86	110	4	115, 116, 118, 120
71	3	72, 86, 118	90	3	99, 102, 103	111	4	112, 115, 116, 120
69	3	88, 98, 114	51	2	89, 113	11	3	12, 59, 61
41	3	64, 76, 96	54	2	77, 110	20	3	35, 74, 108
23	3	43, 84, 114	60	2	83, 95	41	3	45, 61, 82
8	3	103, 114, 120	63	2	73, 97	46	3	58, 64, 107
115	2	119, 120	84	2	99, 101	79	3	80, 82, 97
113	2	116, 117	85	2	114, 120	91	3	97, 108, 117
110	2	112, 115	89	2	113, 117	92	3	99, 105, 110
98	2	103, 110	95	2	104, 108	103	3	109, 110, 119
93	2	96, 106	104	2	106, 108	109	3	110, 112, 118
89	2	113, 117	106	2	108, 119	58	2	101, 109
88	2	103, 110	110	2	112, 115	61	2	68, 84
85	2	114, 120	112	2	118, 119	64	2	78, 104
80	2	81, 86	113	2	116, 117	65	2	66, 107
77	2	100, 105	115	2	119, 120	69	2	92, 104
60	2	83, 95	8	1	114	80	2	88, 98
51	2	113, 117	73	1	109	87	2	97, 115
43	2	109, 118	79	1	121	90	2	115, 120
35	2	46, 74	81	1	104	104	2	111, 112
116	1	117	82	1	86	107	2	112, 120
108	1	109	86	1	114	113	2	114, 121
105	1	118	88	1	103	116	2	118, 120
104	1	106	91	1	114	32	1	62
91	1	114	98	1	103	50	1	65
86	1	114	100	1	112	56	1	99
84	1	101	107	1	119	63	1	82
82	1	86	108	1	109	75	1	114
81	1	104	109	1	119	76	1	96
79	1	121	116	1	117	81	1	82
73	1	109	78	0	-	83	1	98
63	1	97	87	0	-	88	1	91
121	0	-	93	0	-	99	1	105
120	0	-	94	0	-	108	1	110
119	0	-	97	0	-	112	1	120
118	0	-	101	0	-	114	1	121
117	0	-	103	0	-	115	1	118
114	0	-	105	0	-	118	1	119
111	0	-	111	0	-	84	0	-
107	0	-	114	0	-	98	0	-
103	0	-	117	0	-	102	0	-
101	0	-	118	0	-	117	0	-
97	0	-	119	0	-	119	0	-
94	0	-	120	0	-	120	0	-
87	0	-	121	0	-	121	0	-

The comparisons among groups are displayed in each column (H vs BC, H vs BBP and BBP vs BC). The numbers of missing links in each node are ordered by their magnitude from the highest to the lowest. Also it is possible to identify the links missing in each node.

The greatest amount of disconnections by node in the three groups is given in Table 3. It is noteworthy that it is node 24, which is the most disconnected, both in the H vs BC groups (27 disconnections) as in BBP vs BC (33 disconnections). This is remarkable since node 24 is a hub in both networks (H and BBP) (Table 3). A similar pattern was observed in the MCF10 Networks (S2 Table) when the H and BC groups were compared.

It is also important to note that node 11 is the most disconnected (19 lost links) in BBP with respect to the network of H (Table 4). There are also some nodes that do not lose any links among networks while some are conserved in the three groups of women (nodes 117, 119, 120, 121).

## Discussion

No disease exists in isolation [33]. That is, given the functional interdependencies among the molecular components in a human cell, a disease is rarely a consequence of an abnormality in a single gene, but reflects the perturbations of the complex intracellular network [34]. Cancer contains many manifestations of networks at various levels of organization, including the genetic [35,36], cellular [37,38], and phenotypic [39]. The emerging tools of network medicine offer a platform to explore systematically not only the molecular complexity of a particular disease, leading to the identification of disease modules and pathways, but also the molecular relationships between apparently distinct pathological phenotypes [34].

Our results are compatible with the hypothesis of there being a complex immunological network interconnecting the IgG antibody producing clones against at least 121 protein antigens (nodes) differing in their molecular weights (MW). Although the networks of the different groups of women differed in their total numbers of links, the numeric differences were not found to be statistically significant (P = 0.207). This lack of differences among networks could be explained by the shared protein motifs or immunological cross-reactivity, in a manner in which the number of nodes tend to equality and differences become not statistically significant. However, the connectivity intensities [I = NxM] of the association) of the 121 nodes in each of the networks (H, BBP and BC) were statistically different (P = 0.001). That is, the differences found in breast pathology (BBP and BC), and particularly in BC, may be due to the connectivity of the network rather than to the number of nodes in each network.

But there were not only differences in the network connectivity among groups, the number of the most connected nodes (hubs) decreases with the disease, since the diminishing of the nodes having 19 to 24 links were more pronounced in the groups of women having some pathology (Fig. 2). As such, the loss of connectivity in the BC group is probably the consequence of the loss of connectivity of the hubs, as has been seen in other networks [39, 40]. More importantly, by way of the disconnection analysis performed among networks, it was possible to identify the lost node in the BC group (node 24), which is the most connected hub in the H network. This striking result makes node 24 a potentially therapeutic target. Similarly, when the MCF10 Networks were analyzed, we found that the most connected node of the H network (node 111) was also one of the most disconnected nodes in BC (S1 Table and S2 Table).

Nonetheless, since the antigens’ characterization is currently being carried out by our group, it is yet not possible to assign any function to the missing nodes’ links. However, as for the possible actors and events involved in the immunological control of cancer cells, one may state: Complement (C) fixing antibodies reacting with antigens on the surface of the cancer cells and drilling lethal holes in the cells’ membranes, as well as the recruiting into the site of the tumor immunologically active natural killer cells and macrophages, which exert their noxious action though increasing their activation and promoting their production of hydrogen peroxide, superoxide, the hydroxyl radical and singlet oxygen, altogether key elements which can deliver a deleterious impact upon the membranes of the tumor’s cells, thus limiting their reproduction and, in fact, killing many of them [30, 32–43].

As for the network’s state of arousal of their hubs, nodes and links, which seems to provide protection against BC, as well as the expectation of an effective vaccine, one would anticipate that, at least, be the same which activates against a preexisting BC, and which appears to inflict so much damage to it, or that its strength be increased and/or enriched by incorporating other actors or combining them in a different manner, more effective and durable, as do other networks that are biologically active such as: the networks of neurons and with the striated muscles in the neuromuscular plaques; the networks of mutualist symbiont plants such as beans and corn [44]; the network of flocks of birds migrating southbound [45]; the network of scouting ants weaving trails that lead thousands of sister ants to the leaves of trees and take them back to their specific anthill; the group of foraging gazelles while being guarded against predators by their fellow vigilantes connected to the group by means of a communicative system consisting of a certain pattern of ground waves created by their hooves kicking the ground; that of the cancer genes in cancer patients [46].

## Supporting Information

S1 Table Classification of Immunological Bands of the MCF10 cell-line, according to the Number (N) of Variables with which each one of them significantly correlated (P<0.05), the Average Magnitude (M) of the correlations, the Connection Intensity (I = NxM) it contributes to the Network and its Intensity Ranking Order (R). (DOCX)

S2 Table MCF10 Network Disconnections. A comparison between groups are display in each column (H vs BC, H vs BBP and BBP vs BC). The number of missed links in each node are ordered by their magnitude from the highest to the lowest. Also it is possible to visualize the links missed in each node. Bolded and underlined are the most connected Nodes ranked from 1 to 10.
(DOCX)
