# Supplementary material for: The Network of Antigen-Antibody Reactions in Adult Women with Breast Cancer or Benign Breast Pathology or without Breast Pathology
Source: PLoS One. 2015 Mar 17;10(3):e0119014. doi: 10.1371/journal.pone.0119014 (PMC4363365; doi:10.1371/journal.pone.0119014)
Supplement: S1 Table — (DOCX) [file pone.0119014.s001.docx]

**S1 Table.** Classification of Immunological Bands of the MCF10 cell-line, according to the Number (N) of Variables with which each one of them significantly correlated (P<0.05), the Average Magnitude (M) of the correlations, the Connection Intensity (I = NxM) it contributes to the Network and its Intensity Ranking Order (R). Bolded and underlined are the most connected Nodes ranked from 1 to 10.

| **Variable** | **BC** | | | | **BBP** | | | | **H** | | | |
| --- | --- | --- | --- | --- | --- | --- | --- | --- | --- | --- | --- | --- |
|  | **N** | **M** | **I** | **R** | **N** | **M** | **I** | **R** | **N** | **M** | **I** | **R** |
| **111** | 0 | 0.00 | 0.00 | 101 | 0 | 0.00 | 0.00 | **105** | 8 | 0.70 | 5.62 | **1** |
| **95** | 0 | 0.00 | 0.00 | **93** | 2 | 0.77 | 1.54 | 21 | 8 | 0.50 | 4.01 | **2** |
| **107** | 7 | 0.42 | 2.95 | 13 | 4 | 0.31 | 1.22 | **26** | 6 | 0.60 | 3.59 | **3** |
| **65** | 5 | 0.63 | 3.15 | 12 | 5 | 0.60 | 2.98 | **6** | 6 | 0.56 | 3.38 | **4** |
| **86** | 5 | 0.46 | 2.29 | 19 | 4 | 0.58 | 2.31 | **14** | 6 | 0.54 | 3.25 | **5** |
| **76** | 7 | 0.52 | 3.65 | 7 | 6 | 0.41 | 2.46 | 11 | 6 | 0.54 | 3.25 | **6** |
| **94** | 0 | 0.00 | 0.00 | 92 | 0 | 0.00 | 0.00 | 94 | 5 | 0.62 | 3.10 | **7** |
| **92** | 10 | 0.35 | 3.48 | 10 | 9 | 0.48 | 4.30 | 3 | 5 | 0.59 | 2.95 | **8** |
| **81** | 7 | 0.24 | 1.69 | 26 | 4 | 0.61 | 2.45 | **12** | 4 | 0.71 | 2.84 | **9** |
| **98** | 12 | 0.33 | 4.01 | 3 | 5 | 0.35 | 1.77 | 19 | 4 | 0.71 | 2.84 | **10** |
| **73** | 6 | 0.53 | 3.17 | 11 | 4 | 0.52 | 2.09 | 17 | 6 | 0.45 | 2.70 | 11 |
| **108** | 6 | 0.35 | 2.13 | 20 | 6 | 0.61 | 3.68 | 4 | 7 | 0.37 | 2.58 | 12 |
| **91** | 6 | 0.62 | 3.73 | 6 | 7 | 0.40 | 2.81 | 8 | 5 | 0.47 | 2.35 | 13 |
| **70** | 1 | -0.69 | -0.69 | 108 | 3 | 0.21 | 0.64 | 33 | 4 | 0.56 | 2.24 | 14 |
| **55** | 4 | 0.46 | 1.84 | 24 | 5 | 0.58 | 2.91 | 7 | 4 | 0.50 | 2.02 | 15 |
| **29** | 0 | 0.00 | 0.00 | **76** | 0 | 0.00 | 0.00 | 73 | 3 | 0.67 | 2.00 | 16 |
| **78** | 3 | 0.61 | 1.82 | 25 | 1 | 0.54 | 0.54 | 37 | 3 | 0.61 | 1.84 | 17 |
| **56** | 4 | 0.40 | 1.60 | 28 | 5 | 0.45 | 2.25 | 15 | 3 | 0.61 | 1.82 | 18 |
| **58** | 3 | 0.24 | 0.71 | 39 | 4 | 0.59 | 2.36 | 13 | 3 | 0.53 | 1.58 | 19 |
| **82** | 5 | 0.50 | 2.50 | 18 | 2 | 0.75 | 1.51 | 24 | 3 | 0.49 | 1.47 | 20 |
| **88** | 3 | 0.55 | 1.64 | **27** | 8 | 0.43 | 3.44 | 5 | 3 | 0.47 | 1.41 | 21 |
| **97** | 7 | 0.42 | 2.92 | **14** | 0 | 0.00 | 0.00 | 95 | 4 | 0.35 | 1.40 | 22 |
| **34** | 0 | 0.00 | 0.00 | 78 | 0 | 0.00 | 0.00 | 76 | 2 | 0.69 | 1.38 | 23 |
| **59** | 2 | 0.38 | 0.76 | 38 | 3 | 0.74 | 2.22 | 16 | 2 | 0.67 | 1.34 | 24 |
| **40** | 4 | 0.28 | 1.12 | 33 | 2 | 0.43 | 0.87 | 30 | 2 | 0.67 | 1.34 | 25 |
| **43** | 2 | 0.56 | 1.13 | **32** | 1 | 0.43 | 0.43 | 43 | 4 | 0.31 | 1.23 | 26 |
| **22** | 0 | 0.00 | 0.00 | 72 | 3 | 0.16 | 0.47 | 40 | 2 | 0.58 | 1.16 | 27 |
| **67** | 5 | 0.72 | 3.61 | 9 | 6 | 0.33 | 1.96 | 18 | 2 | 0.57 | 1.15 | 28 |
| **68** | 13 | 0.30 | 3.96 | 5 | 2 | 0.54 | 1.08 | **27** | 2 | 0.57 | 1.13 | 29 |
| **48** | 4 | -0.01 | -0.03 | 102 | 2 | 0.09 | 0.18 | **45** | 2 | 0.56 | 1.13 | 30 |
| **53** | 1 | 0.46 | 0.46 | 47 | 4 | 0.00 | 0.00 | 82 | 2 | 0.52 | 1.04 | 31 |
| **27** | 1 | 0.69 | 0.69 | 41 | 0 | 0.00 | 0.00 | 71 | 2 | 0.50 | 1.00 | 32 |
| **49** | 5 | 0.73 | 3.63 | 8 | 3 | 0.51 | 1.52 | 22 | 2 | 0.46 | 0.92 | 33 |
| **89** | 7 | 0.36 | 2.53 | 17 | 8 | 0.58 | 4.66 | 1 | 3 | 0.19 | 0.58 | 34 |
| **46** | 1 | 0.50 | 0.50 | 45 | 3 | 0.52 | 1.56 | 20 | 1 | 0.58 | 0.58 | 35 |
| **85** | 4 | -0.02 | -0.08 | 103 | 6 | 0.72 | 4.30 | 2 | 1 | 0.55 | 0.55 | 36 |
| **35** | 2 | 0.47 | 0.95 | **35** | 0 | 0.00 | 0.00 | 77 | 1 | 0.49 | 0.49 | 37 |
| **25** | 0 | 0.00 | 0.00 | 74 | 0 | 0.00 | 0.00 | 70 | 1 | 0.46 | 0.46 | 38 |
| **69** | 3 | -0.21 | -0.64 | **107** | 3 | 0.19 | 0.57 | 35 | 1 | 0.45 | 0.45 | 39 |
| **63** | 4 | 0.52 | 2.08 | 23 | 2 | 0.51 | 1.01 | 29 | 1 | 0.44 | 0.44 | 40 |
| **47** | 3 | 0.10 | 0.29 | 53 | 2 | 0.01 | 0.01 | 47 | 2 | 0.05 | 0.11 | 41 |
| **1** | 0 | 0.00 | 0.00 | 54 | 0 | 0.00 | 0.00 | **48** | 0 | 0.00 | 0.00 | 42 |
| **2** | 0 | 0.00 | 0.00 | 55 | 0 | 0.00 | 0.00 | 49 | 0 | 0.00 | 0.00 | 43 |
| **3** | 1 | 0.46 | 0.46 | 48 | 0 | 0.00 | 0.00 | 50 | 0 | 0.00 | 0.00 | 44 |
| **4** | 0 | 0.00 | 0.00 | 56 | 0 | 0.00 | 0.00 | 51 | 0 | 0.00 | 0.00 | 45 |
| **5** | 0 | 0.00 | 0.00 | 57 | 0 | 0.00 | 0.00 | 52 | 0 | 0.00 | 0.00 | 46 |
| **6** | 0 | 0.00 | 0.00 | 58 | 1 | -1.00 | -1.00 | 110 | 0 | 0.00 | 0.00 | 47 |
| **7** | 0 | 0.00 | 0.00 | 59 | 2 | 0.00 | 0.00 | 53 | 0 | 0.00 | 0.00 | 48 |
| **8** | 0 | 0.00 | 0.00 | 60 | 0 | 0.00 | 0.00 | 54 | 2 | 0.00 | 0.00 | 49 |
| **9** | 0 | 0.00 | 0.00 | 61 | 0 | 0.00 | 0.00 | 55 | 0 | 0.00 | 0.00 | 50 |
| **10** | 0 | 0.00 | 0.00 | 62 | 0 | 0.00 | 0.00 | 56 | 0 | 0.00 | 0.00 | 51 |
| **11** | 0 | 0.00 | 0.00 | 63 | 0 | 0.00 | 0.00 | 57 | 0 | 0.00 | 0.00 | 52 |
| **12** | 0 | 0.00 | 0.00 | 64 | 0 | 0.00 | 0.00 | 58 | 0 | 0.00 | 0.00 | 53 |
| **13** | 0 | 0.00 | 0.00 | 65 | 0 | 0.00 | 0.00 | 59 | 0 | 0.00 | 0.00 | 54 |
| **14** | 0 | 0.00 | 0.00 | 66 | 0 | 0.00 | 0.00 | 60 | 2 | 0.00 | 0.00 | 55 |
| **15** | 0 | 0.00 | 0.00 | 67 | 0 | 0.00 | 0.00 | 61 | 0 | 0.00 | 0.00 | 56 |
| **16** | 0 | 0.00 | 0.00 | 68 | 0 | 0.00 | 0.00 | 62 | 0 | 0.00 | 0.00 | 57 |
| **17** | 0 | 0.00 | 0.00 | 69 | 0 | 0.00 | 0.00 | 63 | 0 | 0.00 | 0.00 | 58 |
| **18** | 1 | 0.44 | 0.44 | 49 | 0 | 0.00 | 0.00 | 64 | 0 | 0.00 | 0.00 | 59 |
| **19** | 0 | 0.00 | 0.00 | 70 | 0 | 0.00 | 0.00 | 65 | 0 | 0.00 | 0.00 | 60 |
| **20** | 1 | 0.58 | 0.58 | **42** | 0 | 0.00 | 0.00 | 66 | 0 | 0.00 | 0.00 | 61 |
| **21** | 0 | 0.00 | 0.00 | 71 | 0 | 0.00 | 0.00 | 67 | 0 | 0.00 | 0.00 | 62 |
| **23** | 1 | 0.69 | 0.69 | 40 | 0 | 0.00 | 0.00 | 68 | 0 | 0.00 | 0.00 | 63 |
| **24** | 0 | 0.00 | 0.00 | 73 | 0 | 0.00 | 0.00 | 69 | 0 | 0.00 | 0.00 | 64 |
| **26** | 1 | 0.40 | 0.40 | 51 | 1 | 0.66 | 0.66 | 32 | 0 | 0.00 | 0.00 | 65 |
| **28** | 0 | 0.00 | 0.00 | 75 | 0 | 0.00 | 0.00 | 72 | 0 | 0.00 | 0.00 | 66 |
| **30** | 1 | 0.40 | 0.40 | 52 | 1 | 0.43 | 0.43 | 44 | 0 | 0.00 | 0.00 | 67 |
| **31** | 1 | 0.55 | 0.55 | 43 | 3 | 0.48 | 1.44 | 25 | 0 | 0.00 | 0.00 | 68 |
| **32** | 0 | 0.00 | 0.00 | 77 | 0 | 0.00 | 0.00 | 74 | 0 | 0.00 | 0.00 | 69 |
| **33** | 1 | 0.51 | 0.51 | 44 | 2 | 0.00 | 0.00 | 75 | 0 | 0.00 | 0.00 | 70 |
| **36** | 2 | 0.44 | 0.89 | 36 | 0 | 0.00 | 0.00 | 78 | 0 | 0.00 | 0.00 | 71 |
| **37** | 3 | 0.70 | 2.09 | 22 | 1 | 0.54 | 0.54 | 38 | 0 | 0.00 | 0.00 | 72 |
| **38** | 2 | 0.43 | 0.86 | **37** | 1 | 0.43 | 0.43 | 42 | 0 | 0.00 | 0.00 | 73 |
| **39** | 1 | -0.41 | -0.41 | **106** | 1 | -0.59 | -0.59 | 108 | 0 | 0.00 | 0.00 | 74 |
| **41** | 3 | -0.08 | -0.23 | 105 | 3 | 0.24 | 0.71 | 31 | 0 | 0.00 | 0.00 | 75 |
| **42** | 3 | 0.49 | 1.48 | 29 | 1 | 0.47 | 0.47 | **39** | 0 | 0.00 | 0.00 | 76 |
| **44** | 0 | 0.00 | 0.00 | 79 | 0 | 0.00 | 0.00 | 79 | 0 | 0.00 | 0.00 | 77 |
| **45** | 0 | 0.00 | 0.00 | 80 | 0 | 0.00 | 0.00 | 80 | 0 | 0.00 | 0.00 | 78 |
| **50** | 2 | 0.57 | 1.13 | 31 | 0 | 0.00 | 0.00 | 81 | 0 | 0.00 | 0.00 | 79 |
| **51** | 1 | 0.46 | 0.46 | 46 | 1 | 0.45 | 0.45 | 41 | 0 | 0.00 | 0.00 | 80 |
| **52** | 4 | -0.03 | -0.13 | 104 | 2 | -0.03 | -0.05 | 107 | 0 | 0.00 | 0.00 | 81 |
| **54** | 4 | 0.29 | 1.18 | 30 | 0 | 0.00 | 0.00 | 83 | 0 | 0.00 | 0.00 | 82 |
| **57** | 2 | -0.45 | -0.90 | 109 | 1 | -0.62 | -0.62 | 109 | 0 | 0.00 | 0.00 | 83 |
| **61** | 0 | 0.00 | 0.00 | 81 | 0 | 0.00 | 0.00 | 84 | 0 | 0.00 | 0.00 | 84 |
| **62** | 0 | 0.00 | 0.00 | 82 | 0 | 0.00 | 0.00 | 85 | 0 | 0.00 | 0.00 | 85 |
| **66** | 2 | 0.00 | 0.00 | 83 | 3 | 0.18 | 0.55 | 36 | 0 | 0.00 | 0.00 | 86 |
| **71** | 0 | 0.00 | 0.00 | 84 | 0 | 0.00 | 0.00 | 86 | 0 | 0.00 | 0.00 | 87 |
| **74** | 0 | 0.00 | 0.00 | 85 | 0 | 0.00 | 0.00 | 87 | 0 | 0.00 | 0.00 | 88 |
| **75** | 0 | 0.00 | 0.00 | 86 | 0 | 0.00 | 0.00 | 88 | 0 | 0.00 | 0.00 | 89 |
| **77** | 0 | 0.00 | 0.00 | 87 | 6 | 0.42 | 2.50 | 10 | 0 | 0.00 | 0.00 | 90 |
| **79** | 0 | 0.00 | 0.00 | 88 | 0 | 0.00 | 0.00 | 89 | 0 | 0.00 | 0.00 | 91 |
| **80** | 0 | 0.00 | 0.00 | 89 | 0 | 0.00 | 0.00 | 90 | 0 | 0.00 | 0.00 | 92 |
| **83** | 0 | 0.00 | 0.00 | 90 | 0 | 0.00 | 0.00 | 91 | 0 | 0.00 | 0.00 | 93 |
| **84** | 0 | 0.00 | 0.00 | 91 | 0 | 0.00 | 0.00 | 92 | 0 | 0.00 | 0.00 | 94 |
| **87** | 5 | 0.53 | 2.64 | 16 | 0 | 0.00 | 0.00 | 93 | 0 | 0.00 | 0.00 | 95 |
| **99** | 0 | 0.00 | 0.00 | 94 | 0 | 0.00 | 0.00 | 96 | 0 | 0.00 | 0.00 | 96 |
| **100** | 0 | 0.00 | 0.00 | 95 | 0 | 0.00 | 0.00 | 97 | 0 | 0.00 | 0.00 | 97 |
| **101** | 0 | 0.00 | 0.00 | 96 | 0 | 0.00 | 0.00 | 98 | 0 | 0.00 | 0.00 | 98 |
| **102** | 0 | 0.00 | 0.00 | 97 | 0 | 0.00 | 0.00 | 99 | 0 | 0.00 | 0.00 | 99 |
| **103** | 0 | 0.00 | 0.00 | 98 | 0 | 0.00 | 0.00 | 100 | 0 | 0.00 | 0.00 | 100 |
| **104** | 0 | 0.00 | 0.00 | 99 | 0 | 0.00 | 0.00 | 101 | 0 | 0.00 | 0.00 | 101 |
| **105** | 0 | 0.00 | 0.00 | 100 | 0 | 0.00 | 0.00 | 102 | 0 | 0.00 | 0.00 | 102 |
| **106** | 8 | 0.50 | 4.01 | 4 | 3 | 0.85 | 2.54 | 9 | 0 | 0.00 | 0.00 | 103 |
| **109** | 7 | 0.30 | 2.09 | 21 | 0 | 0.00 | 0.00 | 103 | 0 | 0.00 | 0.00 | 104 |
| **110** | 9 | 0.57 | 5.10 | 2 | 0 | 0.00 | 0.00 | 104 | 0 | 0.00 | 0.00 | 105 |
| **112** | 10 | 0.61 | 6.10 | 1 | 0 | 0.00 | 0.00 | **106** | 0 | 0.00 | 0.00 | 106 |
| **96** | 6 | -0.32 | -1.92 | 111 | 3 | -0.48 | -1.44 | 111 | 2 | -0.01 | -0.01 | 107 |
| **64** | 5 | 0.56 | 2.78 | 15 | 3 | 0.51 | 1.52 | 23 | 2 | -0.07 | -0.14 | 108 |
| **60** | 2 | 0.55 | 1.11 | 34 | 3 | 0.19 | 0.58 | 34 | 2 | -0.15 | -0.30 | 109 |
| **72** | 1 | 0.42 | 0.42 | 50 | 2 | 0.05 | 0.11 | 46 | 3 | -0.17 | -0.52 | 110 |
| **90** | 5 | -0.52 | -2.59 | 112 | 8 | -0.45 | -3.64 | 112 | 1 | -0.58 | -0.58 | 111 |
| **93** | 2 | -0.48 | -0.95 | 110 | 2 | 0.51 | 1.01 | 28 | 1 | -0.73 | -0.73 | 112 |
| **Totals** | 259 | 21.93 | 92.76 |  | 188 | 18.20 | 68.75 |  | 156 | 19.76 | 72.41 |  |
