# Supplementary material for: The Network of Antigen-Antibody Reactions in Adult Women with Breast Cancer or Benign Breast Pathology or without Breast Pathology
Source: PLoS One. 2015 Mar 17;10(3):e0119014. doi: 10.1371/journal.pone.0119014 (PMC4363365; doi:10.1371/journal.pone.0119014)
Supplement: S2 Table — A comparison between groups are display in each column (H vs BC, H vs BBP and BBP vs BC). The number of missed links in each node are ordered by their magnitude from the highest to the lowest. Also it is possible to visualize the links missed in each node. Bolded and underlined are the most connected Nodes ranked from 1 to 10. (DOCX) [file pone.0119014.s002.docx]

**S2 Table. MCF10 Network Disconnections**. A comparison between groups are display in each column (H vs BC, H vs BBP and BBP vs BC). The number of missed links in each node are ordered by their magnitude from the highest to the lowest. Also it is possible to visualize the links missed in each node.

| **H vs BC** | | | **H vs BBP** | | | **BBP vs BC** | | |
| --- | --- | --- | --- | --- | --- | --- | --- | --- |
| **Node** | **Number of disconnections** | **Disconnected nodes** | **Node** | **Number of disconnections** | **Disconnected nodes** | **Node** | **Number of disconnections** | **Disconnected nodes** |
| **95** | 8 | 7,8,14,23,48,59,76,91 | **95** | 8 | 7,8,14,23,48,59,78,91 | **92** | 8 | 37,58,59,64,77,85,86,91 |
| **108** | 7 | 14,27,29,56,72,73,91 | **108** | 7 | 14,27,29,56,72,73,92 | **88** | 7 | 5,6,25,37,55,59,85 |
| **111** | 7 | 11,22,56,58,73,86,108 | **111** | 7 | 11,22,56,58,73,86,108 | **56** | 6 | 36,39,40,41,49,53 |
| **86** | 6 | 22,27,29,47,58,70 | **107** | 6 | 8,34,43,81,89,95 | **77** | 6 | 3,26,33,43,67,72 |
| **107** | 6 | 8,34,43,81,89,97 | **65** | 5 | 25,48,49,53,55 | **89** | 6 | 26,49,63,70,78,82 |
| **65** | 5 | 25,48,49,53,55 | **76** | 5 | 10,43,48,60,70 | **91** | 6 | 3,20,26,42,72,73 |
| **76** | 5 | 10,43,48,60,70 | **86** | 5 | 22,27,29,47,70 | **108** | 6 | 3,31,36,40,63,65 |
| **55** | 4 | 10,20,35,40 | **43** | 4 | 7,8,14,23 | **67** | 5 | 2,26,49,52,533 |
| **70** | 4 | 18,22,51,58 | **73** | 4 | 2,27,29,72 | **90** | 5 | 26,49,52,53,77 |
| **92** | 4 | 18,47,70,86 | **91** | 4 | 7,8,43.58 | **96** | 5 | 20,33,35,49,92 |
| **94** | 4 | 23,25,35,53 | **92** | 4 | 18,22,47,70 | **53** | 4 | 5,6,7,49 |
| **43** | 3 | 7,14,23 | **94** | 4 | 23,25,35,53 | **58** | 4 | 3,4,22,43 |
| **48** | 3 | 10,14,43 | **48** | 3 | 10,14,43 | **64** | 4 | 37,49,58,59 |
| **58** | 3 | 22,27,29 | **55** | 3 | 10,20,40 | **76** | 4 | 5,6,22,31 |
| **72** | 3 | 3,56,66 | **56** | 3 | 2,27,29 | **81** | 4 | 35,42,64,73 |
| **73** | 3 | 2,27,29 | **70** | 3 | 18,22,58 | **85** | 4 | 25,55,64,68 |
| **78** | 3 | 49,57,63 | **72** | 3 | 3,56,66 | **86** | 4 | 39,43,58,77 |
| **79** | 3 | 3,8,49 | **78** | 3 | 49,57,63 | **107** | 4 | 30,72,82,98 |
| **82** | 3 | 33,35,40 | **81** | 3 | 3,8,49 | **22** | 3 | 3,5,6 |
| **88** | 3 | 10,11,65 | **82** | 3 | 33,35,40 | **31** | 3 | 3,20,22 |
| **89** | 3 | 34,52,,81 | **88** | 3 | 10,11,65 | **60** | 3 | 4,46,48 |
| **91** | 3 | 7,8,43 | **89** | 3 | 34,52,81 | **70** | 3 | 51,63,66 |
| **98** | 3 | 49,57,63 | **97** | 3 | 7,51,85 | **73** | 3 | 35,42,47 |
| **14** | 2 | 7,8 | **98** | 3 | 49,57,63 | **98** | 3 | 38,58,91 |
| **22** | 2 | 11,18 | **8** | 2 | 3, 7 | **7** | 2 | 5,6 |
| **29** | 2 | 11,22 | **14** | 2 | 7,8 | **33** | 2 | 5,6 |
| **34** | 2 | 3,8 | **22** | 2 | 11,18 | **40** | 2 | 4,36 |
| **40** | 2 | 20,35 | **27** | 2 | 11,22 | **43** | 2 | 2,4 |
| **49** | 2 | 10,34 | **29** | 2 | 11,22 | **46** | 2 | 2,30 |
| **53** | 2 | 10,25 | **34** | 2 | 3,8 | **49** | 2 | 2,30 |
| **59** | 2 | 2,43 | **40** | 2 | 20,35 | **52** | 2 | 46,47 |
| **63** | 2 | 26,57 | **41** | 2 | 8,33 | **55** | 2 | 35,48 |
| **64** | 2 | 7,51 | **49** | 2 | 10,34 | **63** | 2 | 3,31 |
| **69** | 2 | 11,56 | **53** | 2 | 10,25 | **65** | 2 | 3,20 |
| **96** | 2 | 22,85 | **58** | 2 | 27,29 | **66** | 2 | 25,63 |
| **97** | 2 | 7,85 | **59** | 2 | 2,43 | **69** | 2 | 26,60 |
| **8** | 1 | 7 | **63** | 2 | 26,57 | **82** | 2 | 30,41 |
| **25** | 1 | 10 | **64** | 2 | 7,51 | **93** | 2 | 39,60 |
| **27** | 1 | 11 | **67** | 2 | 3,22 | **26** | 1 | 2 |
| **42** | 1 | 26 | **68** | 2 | 49,58 | **30** | 1 | 2 |
| **46** | 1 | 2 | **69** | 2 | 11,56 | **37** | 1 | 25 |
| **56** | 1 | 28 | **96** | 2 | 22,85 | **38** | 1 | 35 |
| **60** | 1 | 7 | **25** | 1 | 10 | **39** | 1 | 20 |
| **67** | 1 | 3 | **35** | 1 | 25 | **41** | 1 | 30 |
| **68** | 1 | 58 | **60** | 1 | 7 | **42** | 1 | 26 |
| **85** | 1 | 64 | **90** | 1 | 3 | **48** | 1 | 47 |
| **93** | 1 | 46 | **93** | 1 | 46 | **51** | 1 | 20 |
| **1** | 0 | 0 | **1** | 0 | 0 | **57** | 1 | 56 |
| **2** | 0 | 0 | **2** | 0 | 0 | **59** | 1 | 55 |
| **3** | 0 | 0 | **3** | 0 | 0 | **72** | 1 | 46 |
| **4** | 0 | 0 | **4** | 0 | 0 | **78** | 1 | 23 |
| **5** | 0 | 0 | **5** | 0 | 0 | **1** | 0 | 0 |
| **6** | 0 | 0 | **6** | 0 | 0 | **2** | 0 | 0 |
| **7** | 0 | 0 | **7** | 0 | 0 | **3** | 0 | 0 |
| **9** | 0 | 0 | **9** | 0 | 0 | **4** | 0 | 0 |
| **10** | 0 | 0 | **10** | 0 | 0 | **5** | 0 | 0 |
| **11** | 0 | 0 | **11** | 0 | 0 | **6** | 0 | 0 |
| **12** | 0 | 0 | **12** | 0 | 0 | **8** | 0 | 0 |
| **13** | 0 | 0 | **13** | 0 | 0 | **9** | 0 | 0 |
| **15** | 0 | 0 | **15** | 0 | 0 | **10** | 0 | 0 |
| **16** | 0 | 0 | **16** | 0 | 0 | **11** | 0 | 0 |
| **17** | 0 | 0 | **17** | 0 | 0 | **12** | 0 | 0 |
| **18** | 0 | 0 | **18** | 0 | 0 | **13** | 0 | 0 |
| **19** | 0 | 0 | **19** | 0 | 0 | **14** | 0 | 0 |
| **20** | 0 | 0 | **20** | 0 | 0 | **15** | 0 | 0 |
| **21** | 0 | 0 | **21** | 0 | 0 | **16** | 0 | 0 |
| **23** | 0 | 0 | **23** | 0 | 0 | **17** | 0 | 0 |
| **24** | 0 | 0 | **24** | 0 | 0 | **18** | 0 | 0 |
| **26** | 0 | 0 | **26** | 0 | 0 | **19** | 0 | 0 |
| **28** | 0 | 0 | **28** | 0 | 0 | **20** | 0 | 0 |
| **30** | 0 | 0 | **30** | 0 | 0 | **21** | 0 | 0 |
| **31** | 0 | 0 | **31** | 0 | 0 | **23** | 0 | 0 |
| **32** | 0 | 0 | **32** | 0 | 0 | **24** | 0 | 0 |
| **33** | 0 | 0 | **33** | 0 | 0 | **25** | 0 | 0 |
| **35** | 0 | 35 | **36** | 0 | 0 | **27** | 0 | 0 |
| **36** | 0 | 0 | **37** | 0 | 0 | **28** | 0 | 0 |
| **37** | 0 | 0 | **38** | 0 | 0 | **29** | 0 | 0 |
| **38** | 0 | 0 | **39** | 0 | 0 | **32** | 0 | 0 |
| **39** | 0 | 0 | **42** | 0 | 0 | **34** | 0 | 0 |
| **41** | 0 | 0 | **44** | 0 | 0 | **35** | 0 | 0 |
| **44** | 0 | 0 | **45** | 0 | 0 | **36** | 0 | 0 |
| **45** | 0 | 0 | **46** | 0 | 0 | **44** | 0 | 0 |
| **47** | 0 | 0 | **47** | 0 | 0 | **45** | 0 | 0 |
| **50** | 0 | 0 | **50** | 0 | 0 | **47** | 0 | 0 |
| **51** | 0 | 0 | **51** | 0 | 0 | **50** | 0 | 0 |
| **52** | 0 | 0 | **52** | 0 | 0 | **54** | 0 | 0 |
| **54** | 0 | 0 | **54** | 0 | 0 | **61** | 0 | 0 |
| **57** | 0 | 0 | **57** | 0 | 0 | **62** | 0 | 0 |
| **61** | 0 | 0 | **61** | 0 | 0 | **68** | 0 | 0 |
| **62** | 0 | 0 | **62** | 0 | 0 | **71** | 0 | 0 |
| **66** | 0 | 0 | **66** | 0 | 0 | **74** | 0 | 0 |
| **71** | 0 | 0 | **71** | 0 | 0 | **75** | 0 | 0 |
| **74** | 0 | 0 | **74** | 0 | 0 | **79** | 0 | 0 |
| **75** | 0 | 0 | **75** | 0 | 0 | **80** | 0 | 0 |
| **77** | 0 | 0 | **77** | 0 | 0 | **83** | 0 | 0 |
| **80** | 0 | 0 | **79** | 0 | 0 | **84** | 0 | 0 |
| **81** | 0 | 0 | **80** | 0 | 0 | **87** | 0 | 0 |
| **83** | 0 | 0 | **83** | 0 | 0 | **94** | 0 | 0 |
| **84** | 0 | 0 | **84** | 0 | 0 | **95** | 0 | 0 |
| **87** | 0 | 0 | **85** | 0 | 0 | **97** | 0 | 0 |
| **90** | 0 | 0 | **87** | 0 | 0 | **99** | 0 | 0 |
| **99** | 0 | 0 | **99** | 0 | 0 | **100** | 0 | 0 |
| **100** | 0 | 0 | **100** | 0 | 0 | **101** | 0 | 0 |
| **101** | 0 | 0 | **101** | 0 | 0 | **102** | 0 | 0 |
| **102** | 0 | 0 | **102** | 0 | 0 | **103** | 0 | 0 |
| **103** | 0 | 0 | **103** | 0 | 0 | **104** | 0 | 0 |
| **104** | 0 | 0 | **104** | 0 | 0 | **105** | 0 | 0 |
| **105** | 0 | 0 | **105** | 0 | 0 | **106** | 0 | 0 |
| **106** | 0 | 0 | **106** | 0 | 0 | **109** | 0 | 0 |
| **109** | 0 | 0 | **109** | 0 | 0 | **110** | 0 | 0 |
| **110** | 0 | 0 | **110** | 0 | 0 | **111** | 0 | 0 |
| **112** | 0 | 0 | **112** | 0 | 0 | **112** | 0 | 0 |
